# No connection between the level of exposition to statins in the population and the incidence/mortality of acute myocardial infarction: An ecological study based on Sweden's municipalities

**DOI:** 10.1186/1477-5751-10-6

**Published:** 2011-05-24

**Authors:** Staffan Nilsson, Sigvard Mölstad, Catarina Karlberg, Jan-Erik Karlsson, Lars-Göran Persson

**Affiliations:** 1 Division of Community Medicine, Department of Medicine and Health Sciences, Faculty of Health Sciences, Linköping University, S-581 83 Linköping, Sweden; 2 Vikbolandet Health Care Centre, Department of Primary Health Care, County Council of Östergötland, Norrköping, Sweden; 3 Unit of R&D in Primary Care, S-551 85 Jönköping, Sweden; 4 Division of Cardiology, Department of Internal Medicine, Ryhov County Hospital, S-551 85 Jönköping, Sweden

**Keywords:** Myocardial infarction, Incidence, Antilipemic agents, Sweden, Population, Ecological study

## Abstract

**Background:**

Randomised controlled trials have shown an excellent preventive effect of statins on ischemic heart disease. Our objective was to investigate if a relation can be detected between acute myocardial infarction- (AMI) mortality or incidence and statin utilisation, for men and women in different age-groups on a population basis.

**Results:**

The utilisation rate of statins increased almost three times for both men and women between 1998 and 2002. During 1998-2000 the incidence of AMI decreased clearly for men but only slightly for women. Mortality decreased from 1998 to 2002. The change in statin utilisation from 1998 to 2000 showed no correlation to the change in AMI mortality from 2000 to 2002. Statin utilisation and AMI- incidence or mortality showed no correlations when adjusting for socio-economic deprivation, antidiabetic drugs and geographic coordinates.

**Conclusions:**

Despite a widespread and increasing utilisation of statins, no correlation to the incidence or mortality of AMI could be detected. Other factors than increased statin treatment should be analysed especially when discussing the allocation of public resources.

## Background

The premature mortality of cardiovascular disease has been declining the last decades in Sweden as well as in many other countries. This is true regarding acute myocardial infarction (AMI) as well, according to nation wide Swedish statistics of AMI covering the period from 1987 to present [1]. On a population basis, a previous study reported a possible negative correlation between the utilisation of lipid lowering drugs and death in ischemic heart disease 1989 - 1993 in Swedish municipalities [2].

During recent years, the statin utilisation has continued to increase and reliable AMI incidence data on a municipality level has become available [3]. Randomised controlled trials have shown unequivocal benefits of statin treatment [4-6]. A detectable relation between statin utilisation and AMI incidence/mortality on a population basis should be of great interest for decisions about allocation of preventive resources.

The aim of this study was to evaluate if there exists an ecological correlation between AMI mortality/incidence and statin utilisation for men and women in different age groups in Sweden's municipalities.

## Methods

The study included 289 of 290 Swedish municipalities. One municipality was excluded due to missing data. The study includes data from1998 to 2002. The Swedish population 40-79 years old in the year 2000 consisted of 1 926 113 men and 1 995 981 women.

The utilisation of statins, and antidiabetic drugs in 1998-2002 among outpatients, was based on the prescriptions served by The Corporation of Pharmacies in Sweden (Apoteket AB) and expressed in Defined Daily Doses (DDD) per 1000 Inhabitants and Day (TID) [7]. The DDD for simvastatin was 15 mg, atorvastatin 10 mg and pravastatin 20 mg. The DDD for antidiabetic drugs included both insulin and oral drugs.

The number of deaths with AMI (ICD-10 code I21 and I22) as the underlying cause was obtained from The Causes of Death Register at The Swedish Board of Health and Welfare. Data on the incidence, attack rate, of AMI was obtained from The AMI Statistics at The Swedish Board of Health and Welfare, and comprised fatal as well as non-fatal AMIs (ICD-10 code I21 and I22), as main or secondary diagnosis [3]. The cut off level of Cardiac troponin T, troponin I or creatine kinase (CK-MB) for AMI was changed in 2001 and therefore more AMIs were diagnosed [8]. Routine coronary revascularisation in unstable coronary artery disease has been shown to reduce mortality and non-fatal myocardial infarctions after one year [9]. The number of persons being subjected to coronary revascularisation i.e. coronary artery by pass grafting and/or percutan coronary intervention was obtained from the Centre of Epidemiology, Swedish Board of Health and Welfare and the Swedish Coronary Angiography and Angioplasty Registry (SCAAR). The yearly incidence and mortality of myocardial infarction and coronary revascularisation rates were calculated for each of the 289 Swedish municipalities for men and women and each of the age groups 40-49, 50-59, 60-69 and 70-79 years. The population sizes for the year 2000 were used.

A socio-economic municipality deprivation index consisting of standardised education level (A), salary (B) and unemployment (C) was calculated for men and women respectively for the year 2000.

For each municipality,

A = (X1 - mean1)/SD1, where X1 is percentage low educated (9 years) in the particular municipality, mean1 is mean percentage of low educated in all municipalities, SD1 is standard deviation of low educated in all municipalities,

B = (X2 - mean2)/SD2, where X2 is percentage having an income within the lowest quartile for Sweden in the particular municipality, mean2 is mean percentage of having an income within the lowest quartile for Sweden in all municipalities, SD2 is standard deviation for having an income within the lowest quartile for Sweden in all municipalities, and

C = (X3 - mean3)/SD3, where X3 is percentage unemployed, 40-59 years old, mean3 is mean percentage of unemployed 40-59 years old in all municipalities, SD3 is standard deviation for unemployed 40-59 years old in all municipalities.

Deprivation index is the sum of A, B and C for the particular municipality [10]. Data on low education and low salary was gathered from Statistics Sweden and on unemployment from The National Labour Market Board.

Data on the geographic x- and y- coordinates of each municipality was obtained from The National Land Survey of Sweden [11,12].

An official grouping of Swedish municipalities into nine groups according to number of inhabitants and infrastructure was used, in order to form subgroups of similar and enough populated municipalities [13]. The groups were 1: Big city (n = 3). Municipalities with a population in excess of 200 000 inhabitants, 2: Suburban municipality (n = 36), 3: Larger town (n = 26). Municipalities with 50 000 to 200 000 inhabitants, 4: Medium-sized town (n = 40). Municipalities with 20 000 to 50 000 inhabitants, 5: Industrial municipality (n = 53), 6: Rural municipality (n = 30), 7: Sparsely populated municipality (n = 29). Municipalities with fewer than 20 000 inhabitants, 8: Other larger municipality (n = 31). Municipalities with 15 000 to 50 000 inhabitants, 9: Other smaller municipality (n = 42). Municipalities with fewer than 15 000 inhabitants.

### Statistical methods

A simple bivariate Pearson correlation coefficient for statin utilisation vs. AMI-incidence and AMI-mortality was calculated for each of the years 1998-2002 and for respective age-groups and gender. Linear regression analysis was used. AMI-incidence was used as the dependent variable and utilisation of statins and antidiabetic drugs, deprivation index, and geographic x- and y-coordinates for each of the 289 municipalities as independent variables. Separate analyses were made for each of the years 1998-2002, and for respective age-groups and gender. The independent variables were ranked in order of significant outcomes vs. incidence in a univariate analyse. According to the ranking, a multivariate statistical model was constructed including the independent variables in the following order, deprivation index, antidiabetic drugs, statin utilisation, x- and y-coordinates. The multivariate model was used in analysing AMI-incidence vs. statin utilisation.

In order to minimise the effect of unusual events and small populations, a multivariate analyses of statin utilisation vs. incidence and mortality, was performed in a group of 26 larger towns, i.e. municipality group 3, with 1857 to 4720 men aged 70-79 years. Considering the time delay for the preventive effect of statins the change in statin utilisation from 1998 to 2000 was estimated as the quotient between statin DDDs per TID in 2000 and in 1998. This quotient was calculated for men aged 70-79 years in each of 149 municipalities, municipality groups 3, 4, 5 and 6. Equivalently, the change in mortality from 2000 to 2002 was calculated in each of those 149 municipalities. A value > 1 implies an increase and < 1 a decrease in statin utilisation or mortality. Subsequently, the quotients representing the change in statin utilisation and the change in AMI mortality were plotted against each other.

The utilisation of statins, AMI-mortality, AMI-incidence and coronary revascularisation rates are shown as means (range) in tables. In the text standard deviations (± SD) also are given.

## Results

### Statins

**Figure 1:**
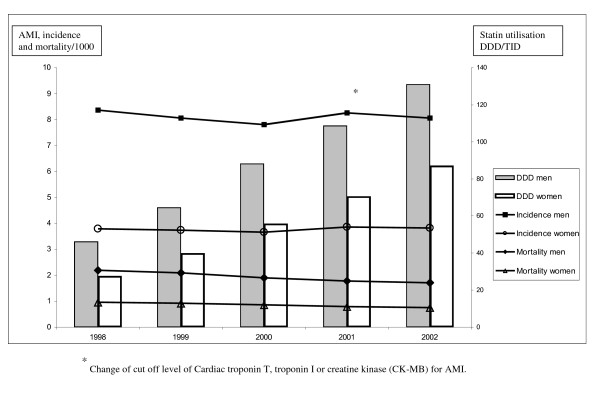
**Incidence and mortality of acute myocardial infarction (AMI) and statin utilisation in the Swedish population, 40-79 years old, 1998-2002**. Utilisation of statins expressed in Defined Daily Doses per 1000 Inhabitants and Day (DDD/TID).

The mean utilisation rate of statins for males increased almost three times, from 46.2 to 131.1 DDD/TID during the five-year period (Figure 1). The highest increase was seen in the oldest age-group (Table 1). In 1998, men aged 60-69 years had the highest use of statins, (mean ± SD) 75 ± 29 DDD/TID, but in 2002 the highest use was observed among men 70-79 years old, 218 ± 57 DDD/TID with a 6-fold variation between municipalities (range: 71-457 DDD/TID). For women, the mean utilisation rate of statins increased more than three times, from 28 to 87 DDD/TID during the five-year period (Figure 1). The highest increase was seen among the oldest (Table 2). In 1998, women aged 60-69 years had the highest use of statins 50 ± 20 DDD/TID but in 2002 the highest use was observed among women aged 70-79 years, 165 ± 47 DDD/TID, with a 6-fold variation between municipalities (range: 56-354 DDD/TID).

**Table 1:** Utilisation of statins, acute myocardial infarction (AMI)-mortality, AMI-incidence and coronary revascularisation rates in Sweden's 289 municipalities' male populations, 1998-2002.

	Utilisation of statins DDD/TID^1^				
	
Age/years	1998	1999	2000	2001	2002
40-49	14.0 (2.28-47.5)	18.2 (2.29-49.5)	23.7 (2.46-59.1)	28.0 (1.63-72.0)	32.0 (4.26-75.7)
50-59	43.4 (10.3-114)	58.2 (12.5-182)	78.1 (20.2-184)	95.0 (34.7-222)	111 (42.5-267)
60-69	75.0 (21.1-196)	103 (34.6-267)	139 (43.1-363)	171 (56.8-391)	202 (93.2-400)
70-79	62.1 (5.82-167)	92.1 (14.9-221)	133 (34.2-319)	174 (47.0-384)	218 (71.3-457)
					
	**AMI-mortality/1000^1^**				
	
**Age/years**	**1998**	**1999**	**2000**	**2001**	**2002**

40-49	0.25(0-3.57)	0.24 (0-4.00)	0.26 (0-6.17)	0.21 (0-2.78)	0.15 (0-3.07)
50-59	0.82 (0-6.05)	0.72 (0-4.67)	0.91 (0-7.50)	0.74 (0-7.25)	0.74 (0-7.14)
60-69	2.94 (0-11.3)	2.65 (0-12.9)	2.58 (0-9.76)	2.36 (0-10.9)	2.40 (0-9.17)
70-79	8.74 (0-24.5)	8.61 (0-24.7)	7.81 (0-25.0)	6.80 (0-23.6)	6.73 (0-24.7)
					
	**AMI-incidence/1000^1^**				
	
**Age/years**	**1998**	**1999**	**2000**	**2001**	**2002**

40-49	1.60 (0-9.67)	1.49 (0-6.22)	1.37 (0-6.17)	1.39 (0-6.02)	1.33 (0-7.75)
50-59	5.00 (0-20.3)	4.53 (0-15.0)	4.63 (0-15.8)	4.93 (0-21.7)	4.63 (0-14.0)
60-69	11.6 (0-27.5)	11.2 (1.3-31.9)	10.7 (0-27.8)	11.1 (0-28.7)	11.6 (0-27.9)
70-79	26.2 (0-61.3)	26.1 (3.0-61.7)	25.6 (4.9-53.7)	25.9 (0-61.4)	25.2 (4.1-54.3)
					
	**Coronary-revascularisation/1000^1^**				
	
**Age/years**	**1998**	**1999**	**2000**	**2001**	**2002**

40-49	1.26 (0-8.00)	1.32 (0-6.17)	1.34 (0-12.9)	1.34 (0-6.30)	1.45 (0-8.10)
50-59	4.00 (0-14.7)	4.27 (0-27.0)	4.41 (0-15.5)	5.14 (0-20.4)	5.21 (0-14.5)
60-69	7.55 (0-19.6)	8.02 (0-24.2)	8.35 (0-20.67)	9.25 (0-32.5)	10.0 (0-26.4)
70-79	8.74 (0-28.5)	9.22 (0-32.5)	9.65 (0-31.9)	10.9 (0-35.6)	11.8 (0-32.5)

Utilisation of statins expressed as Defined Daily Doses per 1000 Inhabitants and Day (DDD/TID).

^1 ^Mean (range)

**Table 2:** Utilisation of statins, acute myocardial infarction (AMI)-mortality, AMI-incidence and coronary revascularisation rates in Sweden's 289 municipalities' female populations, 1998-2002.

	Utilisation of statins DDD/TID^1^				
	
Age/years	1998	1999	2000	2001	2002
40-49	4.98 (0.27-18.7)	6.68 (0.2-32.7)	9.22 (0.13-41.3)	11.2 (0.82-46.4)	13.1 (0.88-38.2)
50-59	19.6 (2.14-59.9)	28.2 (6.45-86.8)	40.3 (11.0-135)	50.6 (15.6-207)	61.5 (20.8-221)
60-69	49.8 (7.52-120)	69.8 (10.7-162)	96.2 (18.1-206)	119 (40.7-251)	141 (45.3-288)
70-79	45.7 (3.05-128)	67.1 (17.4-178)	97.8 (30.9-250)	131 (40.3-315)	165 (56.2-354)
					
	**AMI-Mortality/1000^1^**				
	
**Age/years**	**1998**	**1999**	**2000**	**2001**	**2002**

40-49	0.06 (0-2.56)	0.06 (0-2.36)	0.07 (0-2.60)	0.05 (0-2.56)	0.04 (0-3.36)
50-59	0.15 (0-2.02)	0.17 (0-4.59)	0.22 (0-2.69)	0.21 (0-4.07)	0.19 (0-3.02)
60-69	0.86 (0-8.06)	1.00 (0-7.16)	0.91 (0-7.16)	0.87 (0-7.17	0.75 (0-8.33)
70-79	3.96 (0-15.3)	3.92 (0-17.2)	3.46 (0-13.9)	2.98 (0-13.7)	3.00 (0-20.0)
					
	**AMI-Incidence/1000^1^**				
	
**Age/years**	**1998**	**1999**	**2000**	**2001**	**2002**

40-49	0.45 (0-4.75)	0.46 (0-4.14)	0.41 (0-4.72)	0.46 (0-7.47)	0.42 (0-5.35)
50-59	1.28 (0-10.9)	1.48 (0-5.74)	1.46 (0-8.45)	1.57 (0-11.1)	1.53 (0-6.37)
60-69	4.28 (0-15.4)	4.36 (0-18.0)	4.19 (0-15.7)	4.51 (0-31.7)	4.49 (0-14.9)
70-79	13.1 (0-33.5)	12.8 (0-33.4)	12.6 (0-47.4)	13.0 (0-31.2)	12.8 (0-35.2)
					
	**Coronary-Revascularisation/1000^1^**				
	
**Age/years**	**1998**	**1999**	**2000**	**2001**	**2002**
40-49	0.32 (0-3.36)	0.28 (0-4.14)	0.32 (0-2.14)	0.34 (0-3.30)	0.42 (0-5.35)
50-59	0.95 (0-5.88)	1.13 (0-6.56)	1.24 (0-8.45)	1.28 (0-7.85)	1.25 (0-8.06)
60-69	2.49 (0-11.9)	2.42 (0-15.7)	2.66 (0-12.2)	3.08 (0-13.6)	3.18 (0-11.3)
70-79	3.28 (0-17.2)	3.24 (0-13.1)	3.87 (0-19.1)	3.95 (0-31.9)	4.60 (0-17.4)

Utilisation of statins expressed as Defined Daily Doses per 1000 Inhabitants and Day (DDD/TID).

^1 ^Mean (range)

### Antidiabetic drugs

The mean ± SD utilisation of antidiabetic drugs (DDD/TID) for the male populations, 40-79 years old, increased from 67 ± 38 in 1998 to 87 ± 49 in 2002. For women, the mean utilisation increased from 51 ± 34 1998 to 61 ± 39 in 2002.

### Mortality of AMI

In males, mortality decreased from 2.20 to 1.72/1000, during the five-year period (Figure 1). The largest absolute decrease was among men 70-79 years old and their mean mortality decreased from 8.74/1000 ± 4.42 in 1998 to 6.73/1000 ± 4.01 in 2002 (Table 1). In women, mortality decreased from 0.97 to 0.76/1000, during the five-year period (Figure 1).

### Incidence of AMI

In 2001, the diagnostic criteria for AMI changed. During the first three years of the study the incidence of AMI decreased in males from 8.37 to 7.81/1000 and then again increased to 8.26 in 2001, followed by 8.06 in 2002 (Figure 1). In women, the incidence decreased slightly during the first three years of the study, from 3.79 to 3.66/1000 and then again increased to 3.86 in 2001 and 3.82 in 2002 (Figure 1).

### Coronary revascularisation

Coronary revascularisation rates in males increased during the five-year period from 4.53 to 5.96/1000 in the 40-79 year old population. The highest relative and absolute increase was observed among men 70-79 years old and their mean revascularisation rate increased from 8.74 ± 4.94 in 1998 to 11.8 ± 5.6 in 2002 (Table 1). In women, the coronary revascularisation rates increased during the five-year period from 1.48 to 1.98/1000 in the 40-79 year old population. The highest relative and absolute increase was observed among women 70-79 years old and for those the mean revascularisation rate increased from 3.38 ± 2.60 to 4.60 ± 2.84 in 2002 (Table 2).

### Socio-economic deprivation index

The socio-economic deprivation index for males was (mean ± SD) 0.0 ± 2.01, (range - 6.42- + 6.02) and for females 0.0 ± 2.29 (range -7.5- + 5.2).

### Change in statin utilisation in relation to AMI-mortality change

**Figure 2:**
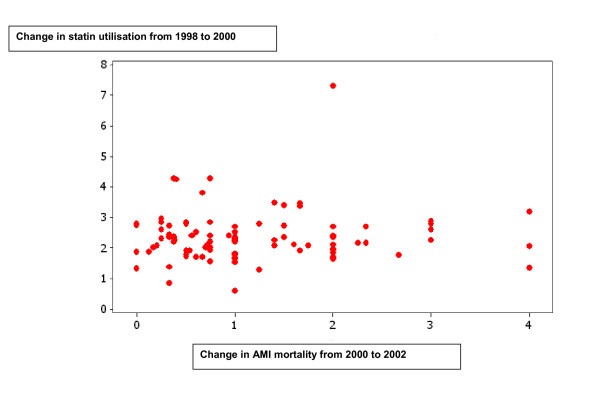
**Change in statin utilisation from 1998 to 2000 plotted against change in acute myocardial infarction (AMI) mortality form 2000 to 2002**. A value > 1 indicates an increase and <1 a decrease. Each dot represents all men, 70-79 years old, in municipality groups 3-6. Municipality groups 3-6 comprises of municipalities enough populated and with a fairly stable infrastructure.

There was no connection between the quotient of statin utilisation in 2000 and 1998 and the quotient of AMI mortality in 2002 and 2000 among the male populations, 70-79 years old in 149 municipalities (Figure 2).

### Bivariate correlation and multivariate analysis

Statin utilisation and AMI -incidence had a statistically significant negative bivariate and multivariate correlation for 70-79 years old men in the three years 1998 to 2000 (Table 3). In 1998 the correlation coefficient (r) was -0.168 (p = 0.004), in 1999 -0.172 (p = 0.003) and in 2000 -0.170 (p = 0.004). The regression coefficient (β) in 1998 was -0.042 (p = 0.043), in 1999 -0.040 (p = 0.014) and in 2000 - 0.026 (p = 0.021). Adjustment was made for socio-economic deprivation, antidiabetic drugs, x- and y-coordinates. For women 70-79 years old, there was a statistically significant negative bivariate correlation 1998-1999. In 1998 r was -0.137 (p = 0.020) and in 1999 -0.154 (p = 0.009). In the multivariate analyses of these years there was a statistical significance only in 1999, β -0.032 (p = 0.011) (Table 3). In the bivariate testing, statin utilisation and AMI-mortality had fewer statistically significant correlations than statin utilisation and AMI-incidence. Multivariate analysis of statin utilisation vs. incidence and vs. mortality of AMI for men 70-79 years old, in 26 larger towns with 50 -200 000 inhabitants showed no statistically significant results.

**Table 3:** Correlation coefficients (r) and regression coefficients (β) for statin utilisation vs. acute myocardial infarction (AMI)-incidence

		Statin utilisation vs. AMI-incidence			
		**Male**		**Female**	

**Age**	**Year**	**r**	**β^1^**	**r**	**β^1^**

40-49	1998	0.051	0.005	0.061	0.009
	1999	0.303***	0.046***	0.165**	0.024*
	2000	0.131*	0.011	0.115	0.011
	2001	0.126*	0.017*	0.040	-0.005
	2002	0.037	0.001	-0.071	-0.008
					

50-59	1998	0.141*	0.007	0.086	0.004
	1999	0.287***	0.028***	0.017	-0.017*
	2000	0.141*	0.011	0.064	-0.002
	2001	-0.047	-0.016**	0.115	0.004
	2002	-0.000	-0.006	0.148*	0.003
					

60-69	1998	0.052	0.010	0.097	-0.0005
	1999	0.026	0.003	0.146*	0.002
	2000	-0.032	-0.001	0.146*	0.010
	2001	0.081	0.009	-0.017	-0.006
	2002	-0.042	-0.004	0.051	-0.004
					

70-79	1998	-0.168**	-0.042*	-0.137*	-0.031
	1999	-0.172**	-0.040*	-0.154**	-0.032*
	2000	-0.170**	-0.026*	-0.042	-0.006
	2001	-0.024	0.005	-0.036	-0.007
	2002	-0.082	-0.009	-0.062	-0.004

^1 ^β for statin utilisation in repeated multivariate linear regression analyses with AMI-incidence as dependent variable and deprivation index, utilisation of antidiabetic drugs, statin utilisation and x- and y-coordinates as independent variables

*P < 0.05, **P < 0.01, ***P < 0.001

## Discussion

From 1998 to 2002 statin utilisation tripled in the Swedish population 40-79 years old. Bivariate and multivariate analysis for different age-groups and gender showed no correlation for statin utilisation vs. AMI incidence or mortality. These ecological observations do not support that statin therapy is a major contributory cause to the decreasing incidence and mortality in AMI.

Results from an ecological study are best not being interpreted at the individual level, thus avoiding the ecological fallacy [2,14]. However, the results can be used as a basis for discussion and for the generation of new alternative hypotheses. The used data collected from different registries were judged to be of sufficient quality [15]. However, we do not know how much of the statins dispensed by the pharmacies in this study that were actually consumed, but adherence with statin therapy is shown to be rather low [16]. The change in diagnostic cut-off values for AMI was an unforeseen draw back, limiting the comparisons with clinical trials and earlier ecological studies. To overcome variations in AMI morbidity and statin utilisation linked to socio-economic factors, we adjusted with a socio-economic deprivation index. Low education and low household income have been shown to be of equal importance for cardiovascular risk for men and women [17,18]. Aiming to adjust for a possible east-west and north-south geographic variation in AMI morbidity in Sweden, adjustment was made for geographical coordinates in each municipality[11,12]. There was a clear increase in coronary revascularisation rates, particularly among men and women 70-79 years old. However, since the indication for revascularisation often is an AMI, this variable was not included in the multivariate model. Data on life-style factors such as smoking, dietary habits or leisure time physical activities were not available on a municipality level. However, changes in life-style factors may be of much higher significance for changes in the incidence of AMI or mortality rates than statins [19,20]. Smoking has gradually decreased in Sweden during the last 30 years both in women and particularly in men and the number of daily smokers in 2005 was 13 and 17 percent for men and women, respectively. To some extent, smoking habits could be assumed to be included in the socio economic deprivation index, since people with low education more often are smokers.

Merlo found a significant reduction of the relative risk of death from ischemic heart disease correlating to a rising utilisation of statins in Swedish municipalities analysed by quartiles 1989-1993 [2]. In our study, in addition to AMI-mortality, we analysed AMI incidence comprised of both lethal and not lethal AMIs, thus using a measure with severe as well as less severe AMIs. We analysed respective age-groups and gender, in contrast to Merlo et al, to investigate a possible correlation between statin utilisation and AMI mortality or AMI-incidence among the oldest with the highest morbidity and utilisation of statins. Our hypothesis was that a possible relationship between statin use and decrease in AMI mortality/incidence would be more likely to be detected in older age groups.

According to randomised controlled trials in both primary and secondary prevention the effects of statins become obvious within 1-2 years after randomisation [5,6]. Considering this time delay we investigated the potential connection between change in statin utilisation and change in mortality two years later among 70 to 79 year old men. One could argue that a connection between a high increase in statin utilisation and a decrease in AMI-mortality on a municipality basis could be more easily detected after a longer period than two years. However, decreased compliance to statin treatment and change in other riskfactors e.g. smoking, obesity or physical exercise would make the results invalid. We don't know how much of the statins that were used for primary or secondary prevention. Increased primary prevention use, potentially results in decreased AMI mortality. Increased secondary prevention use may also result in decreased AMI mortality. However, there is a matter of reversed causation i.e. the more AMIs the more statins used. We addressed this issue by analysing the potential connection between the change in statin utilisation and again change in AMI mortality two years later. The hypothesis was that a big increase in statin utilisation could be related to a decrease in AMI-mortality two years later, irrespective of the indication for statin treatment. However, we found no connection between the change in statin utilisation and again change in AMI mortality two years later (Figure 2).

In this study, no correlations of importance were found between statin utilisation and AMI incidence/mortality (Table 3). It must be emphasised that some of the correlation coefficients in table 3 are significantly different from zero, but the value of the correlation coefficients are very low, so the grade of linearity is non-existent. Significance is only due to a large number of observations. The unequivocal benefits shown in randomised controlled trials are in contrast to the results in this study [4-6]. Number needed to treat (NNT) is the inverted absolute risk reduction. Using data from a primary preventive randomised controlled trial in men [6], would give a NNT/year of 235 (95% CI, 152-490) for non-fatal AMI or death from coronary heart disease. The observed yearly, average increase in male statin use in our study, 21.2 DDD/TID, corresponds to 10.6 study doses of pravastatin and a calculated possible decrease in AMI-incidence of 9/100 000 males during 1998-2000, attributable to statins. In the present study we observed a decrease in AMI incidence of 56/100 000 men, 40-79 year old, between 1998-2000. Hence, a use of statins according to this primary preventive study would theoretically be able to explain 9/56, 16 percent of the observed decrease of AMI-incidence 1998-2000. In a secondary preventive randomised controlled trial [5], the NNT for preventing a mortal AMI/year can be calculated to be 362 (95% CI 227-902). Applying these study data on the present study, statin use might explain 12/48, 25 percent of the observed decrease of male AMI-mortality 1998-2002. Making the same type of theoretical calculation on AMI-incidence data [5], the use of statins should be able to explain 32/56, 57 percent of the observed decrease of male AMI-incidence 1998-2000.

Statin studies are often not planned to reveal possible differences in treatment effects between women and men. No primary preventive reduction of cardiovascular mortality or incidence of non-fatal AMI has been shown in women, but possibly of coronary heart disease events [21]. Secondary preventive effects in women, are less well documented than in men [22]. An interesting finding is that women appeared to be prescribed more statins than men in relation to their risk for AMI, using incidence and mortality as a proxy measure of risk (Figure 1).

## Conclusions

Though a widespread and increasing utilisation of statins, no correlation with AMI incidence/mortality in a general Swedish population, independent of age and gender, could be detected in this explorative study. The benefits shown in clinical trials could not be recognized despite that a high fraction of the population studied used statins. It is obvious that factors other than increased statin treatment should be analysed, especially when discussing the allocation of public resources.

## Abbreviations

AMI, Acute myocardial infarction; β, Regression coefficient; DDD, Defined daily doses; DDD/TID, Defined daily doses per 1000 inhabitants and day; ICD 10, International Classification of Diseases and Related Health Problems 10^th ^version; NNT, Number needed to treat; r, Correlation coefficient.

## Competing interests

JEK has been reimbursed by AstraZeneca, MSD and Pfizer for lectures about statin treatment.

## Authors' contributions

SN carried out the study, participated in the design of the study and wrote the manuscript. SM led the study design and writing. CK did the statistical analyses. JEK and LGP contributed to study design and drafting. All authors read and approved the final manuscript.

## Ethical approval

The study was approved by the ethics committee of the Faculty of Health Sciences of Linköping University.

## Funding

This study was supported by grants from Health Research Council in the South-east of Sweden (FORSS) [grant number 1380] and the County Council of Östergötland. The study was designed, conducted, analysed, and interpreted independently of all funding sources.
